# Removal of a Broken Instrument from a Tooth with Apical Periodontitis Using a Novel Approach

**DOI:** 10.7508/iej.2016.03.018

**Published:** 2016-05-01

**Authors:** Azar Heydari, Mona Rahmani, Mostafa Heydari

**Affiliations:** a Department of Endodontics , Dental School, Shahid Beheshti University of Medical Sciences, Tehran, Iran;; b Dentist, Tehran, Iran

**Keywords:** Apical Surgery, Broken Instrument, Nonsurgical Retreatment

## Abstract

Instrument breakage during treatment can lead to serious complications and carries the risk of treatment failure. When a file breaks in the canal, bypassing or removal can be difficult and the long-term prognosis of the tooth may be compromised. Sometimes surgery may be indicated for removal of the broken segment. Often some part of the root cannot be cleaned because of blockage by the broken file. This report presents a specific approach in non- surgical removal of a broken file from a maxillary lateral incisor with a buccal sinus tract and a broken instrument in the apical third which was partially over extended into the periapical lesion. The broken file was accessed through the sinus tract and pushed into the canal. The canal was cleaned and shaped, filled with mineral trioxide aggregate (MTA).

**Conclusion:**

A sinus tract can be a specific path to reach the root tip and get access to remove the foreign materials pushed beyond the root canal space.

## Introduction

Instrument fracture during root canal therapy (RCT) is a troublesome incident that can interfere with efficient cleaning and shaping of the root canal or act as an irritant to the periapical tissues especially when some part of the separated fragment over extends from the root apex [1-3]. The most common causes of instrument separation include improper or excessive use, inherent physical properties, inadequate access, root canal anatomy and possible manufacturing defects [1, 4]. The prognosis of endodontic treatment of a tooth with a broken instrument in the canal, depends on the stage of instrumentation prior to instrument separation, pretreatment pulpal or periradicular tissue status and whether or not the fractured file can be removed or bypassed [5].

Every attempt should be made for removing the fragment or bypassing it followed by adequate cleaning and shaping and incorporating it into the final canal obturation [6-9]. Sometimes surgery may be needed to remove the broken file and some part of the root that cannot be cleaned because of obstruction created by the broken fragment [10-12]. But surgery caries the risk of injury to the anatomic structures such as the inferior alveolar nerve and/or artery, nasal cavity and maxillary sinus [3, 13]. Moreover, gingival recession, papillae shrinkage and scar tissue formation are frequently seen following apical surgery [12]. Nonsurgical management of periapical lesions has shown a high success rate so it should be considered, if possible, before apical surgery [14, 15].

This report represents the novel approach of non-surgical removal of a separated file fragment through the apical foramen accessed through the apical sinus tract.

## Case Report

A 32-year old male was referred to a private clinic with the chief complaint of recurrent swelling of the upper lip area just under nose and occasional pain and discomfort in periapical area of maxillary right central and lateral incisors.

**Figure 1:**
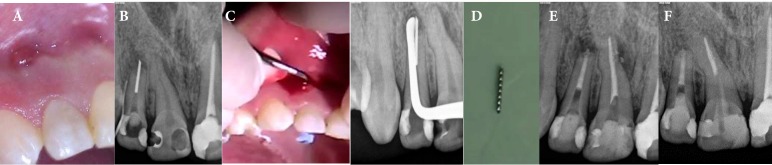
A) Intraoral sinus tract; B) Periapical radiography of the right central and lateral incisors; C) A long shank excavator was passed from the sinus tract to the root tip of the tooth and the broken file was pushed into the canal; D) Retrieved fragment; E) Canal obturated with MTA; F) 18 months after treatment

Intraoral examination revealed a fistula in the buccal vestibule above the lateral incisor (Figure 1A). Clinical examination showed that both right central and lateral incisors had deep composite resin fillings and were slightly tender on percussion and the lateral tooth was sensitive to palpation. Vitality test of both teeth using ENDO-ICE frozen gas (Coltene/Whaledent, Inc., Mahwah, NJ, USA) and electric pulp tester (EPT) (Analytic Technology, Redmond, WA, USA) revealed no response. Dental history showed RCT of the lateral incisor 4 years earlier. Patient reported abscess and sinus tract 6 months after endodontic clinical procedures.

Radiographic examination showed a periapical radiolucency around both incisors (Figure 1B). Apical root resorption of the lateral incisor was evident. A broken instrument was seen in the apical third of the root canal; some part of the segment was over- extended from the canal into the periapical lesion. There was no obturation material or gutta-percha in the canal. The sinus tract was traced using a #30 gutta-percha cone (Dentsply, Maillefer, Ballaigues, Switzerland) and a periapical radiography was taken. The traced gutta-percha reached the root tip of lateral incisor.

The central incisor had no swelling or sinus tract but it had a mild pain in percussion test. Clinical diagnosis was chronic periapical abscess of lateral incisor and chronic apical periodontitis of the central one. After signing the informed consent by the patient, orthograde retreatment and RCT of the lateral and central incisor were planned, respectively.

After administration of local anesthesia using 2% lidocaine containing 1:80000 epinephrine (Darupakhsh, Tehran, Iran) into the buccal vestibule next to the tooth root and also in the palatal mucosa. Access cavity was prepared through the old composite resin restoration. At first removal of the broken fragment by an orthograde approach was applied but releasing the coronal part of the file or negotiating by a K-file was not possible.

Thus, a long shank excavator was passed from the sinus tract to the root tip and the metallic object was sensed (Figure 1C). We determined the position of the excavator, using a periapical radiography (Figure 1D). Then the broken segment was pushed into the canal by the excavator and a periapical radiography was retaken which confirmed the push of the metallic piece into the canal. After application of rubber dam, a #15 K-file (Mani, Tochigi, Japan) was inserted into the canal to bypass the segment. Then, a #30 H-file (Mani, Tochigi, Japan) was inserted into the canal next to the broken fragment and pulled it out (Figure 1E). The working length was determined using a Root ZX apex locator (J. Morita USA, Inc., Irvine, CA, USA). Instrumentation of the canal was performed using K-files (Mani, Tochigi, Japan) and Gates Glidden drills (Mani Inc., Tochigi, Japan) with hybrid preparation technique. Copious irrigation with 5.25% sodium hypochlorite solution (Merck, Darmstadt, Germany) was carried out. After irrigation using normal saline, final rinse was performed using 2% chlorhexidine gluconate (CHX) (Meta Biomed Co., Chung-Ju, Korea). Calcium hydroxide paste (Sultan, Englewood, NS, USA) powder was mixed with 2% CHX to prepare a paste with creamy consistency which was placed into the canal using lentulo spiral. Temporary filling (Cavisol, Golchai, and Tehran, Iran) was placed in the access cavity.

After 2 weeks, the sinus tract had disappeared. After local anesthesia and isolation with rubber dam, calcium hydroxide paste was removed and the canal was irrigated with 2.5% NaOCl and then 2% CHX. The canal was dried using paper points. White ProRoot MTA (Dentsply, Tulsa Dental, Tulsa, OK, USA) was mixed with distilled water according to the manufacturer’s instructions and was placed into the canal with a fine-tipped hand plugger. MTA placement continued till its thickness reached almost 6 mm. Then, a wet paper point (Dentsply Maillefer, Ballaigues, Switzerland) was placed in the canal to expedite MTA setting and the crown was temporarily sealed using Cavisol (Golchai, Tehran, Iran).

The plug’s position was checked using a periapicalradiography (Figure 1F).

The permanent restoration was done using light-cure composite resin one week later. The patient was recalled 6 months later. The buccal sinus tract did not reoccur and the tooth showed no clinical signs/symptoms of recurrent infection or inflammation. Radiographic examination at 6-, 12- and 18- month follow-ups revealed complete healing of the periapical lesion (Figure 1G).

## Discussion

The cause of treatment failure after separation of an endodontic instrument in the root canal, is the clinician’s inability to clean and disinfect the remaining part of the canal due to the impediment [3]. If the instrument cannot be removed or bypassed, maintenance of a fractured instrument in a tooth with a necrotic infected pulp and apical periodontitis, will make the prognosis uncertain. If symptoms persist, apical surgery or extraction should be considered for these cases [16].

The factors determining the potential to remove a separated instrument should be considered during the diagnostic workup. The location of the broken instrument is a major determinant factor [17]. Few studies have reported successful broken file removal from the canal [8, 18].

In the present case, a long segment of a large K-file was broken in the apical part of maxillary incisor. At first bypassing the fragment was tried with a #10 K-file which was not successful and there was no way to retrieve the file by gripping the fragment using braided H-files or K-files and pulling it out [19]. The tip of the file was over extended from the canal into the periapical lesion which might be because of apical root resorption around the fragment. Thus, even if it was possible to bypass the file, a complete apical seal would not be possible. Moreover, the file in the periapex could provoke a foreign body reaction. For this reason, fragment retrieval was tried before indicating surgery.

Several techniques have been introduced for removal of a broken instrument. Masserann’s technique is one of the most current methods for fragment removal [20]. But this method requires vigorous reduction of dentinal walls of the root canal and weakens the root and therefore makes the root susceptible to fracture or root perforation [10, 21]. Surgical removal of the fragment after pushing it out of the apical foramen into the periapex has been reported in some cases [10]. But in the anterior maxilla, gingival recession, including papillae shrinkage and scar tissue formation following apical surgery, can induce aesthetic problems [12].

In the present case, we pushed the fragment from the apex into the canal and successfully extruded it through the coronal part of the canal. As confirmed by the previous studies, periapical lesions localized in the cancellous bone may not be detectable by traditional periapical radiographies unless they involve cortical bone [22, 23]; therefore, the radiolucency around the present tooth in addition to a sinus tract that could be traced by a gutta- percha cone, confirmed that the cortical plate over the tooth had a considerable defect. With this novel approach presented here, an excavator could be passed through the sinus tract to reach the root tip and push the file into the canal.

The apical constriction was destroyed because of apical root resorption. Root canal obturation by MTA could provide a perfect seal [24-27]. So sealing the apical half of the canal with MTA guaranteed profound apical sealing.

## Conclusion

There are different ways to remove a broken instrument from the canal. A sinus tract can be a specific path to reach the root tip and get access to remove the foreign objects/materials provided they are extruded beyond the root canal space.
